# Co-evolution of genomes and plasmids within *Chlamydia trachomatis *and the emergence in Sweden of a new variant strain

**DOI:** 10.1186/1471-2164-10-239

**Published:** 2009-05-21

**Authors:** Helena MB Seth-Smith, Simon R Harris, Kenneth Persson, Pete Marsh, Andrew Barron, Alexandra Bignell, Carina Bjartling, Louise Clark, Lesley T Cutcliffe, Paul R Lambden, Nicola Lennard, Sarah J Lockey, Michael A Quail, Omar Salim, Rachel J Skilton, Yibing Wang, Martin J Holland, Julian Parkhill, Nicholas R Thomson, Ian N Clarke

**Affiliations:** 1 Molecular Microbiology Group, University Medical School, Southampton General Hospital, Southampton, SO16 6YD, UK; 2 The Pathogen Sequencing Unit, The Wellcome Trust Sanger Institute, Hinxton, Cambridge, CB10 1SA, UK; 3 Department of Clinical Microbiology, Malmo University Hospital, SE 205 02 Malmo, Sweden; 4 Health Protection Agency South East, Southampton General Hospital, Southampton, SO16 6YD, UK; 5 Department of Obstetrics and Gynaecology, Malmo University Hospital, SE 205 02, Malmo, Sweden; 6 Viral Diseases Programme, Medical Research Council PO Box 273, Banjul, The Gambia; 7 Clinical Research Unit, Department of Infectious and Tropical Diseases, London School of Hygiene and Tropical Medicine, Keppel Street, London, WC1E 7HT, UK

## Abstract

**Background:**

*Chlamydia trachomatis *is the most common cause of sexually transmitted infections globally and the leading cause of preventable blindness in the developing world. There are two biovariants of *C. trachomatis*: 'trachoma', causing ocular and genital tract infections, and the invasive 'lymphogranuloma venereum' strains. Recently, a new variant of the genital tract *C. trachomatis *emerged in Sweden. This variant escaped routine diagnostic tests because it carries a plasmid with a deletion. Failure to detect this strain has meant it has spread rapidly across the country provoking a worldwide alert. In addition to being a key diagnostic target, the plasmid has been linked to chlamydial virulence. Analysis of chlamydial plasmids and their cognate chromosomes was undertaken to provide insights into the evolutionary relationship between chromosome and plasmid. This is essential knowledge if the plasmid is to be continued to be relied on as a key diagnostic marker, and for an understanding of the evolution of *Chlamydia trachomatis*.

**Results:**

The genomes of two new *C. trachomatis *strains were sequenced, together with plasmids from six *C. trachomatis *isolates, including the new variant strain from Sweden. The plasmid from the new Swedish variant has a 377 bp deletion in the first predicted coding sequence, abolishing the site used for PCR detection, resulting in negative diagnosis. In addition, the variant plasmid has a 44 bp duplication downstream of the deletion. The region containing the second predicted coding sequence is the most highly conserved region of the plasmids investigated. Phylogenetic analysis of the plasmids and chromosomes are fully congruent. Moreover this analysis also shows that ocular and genital strains diverged from a common *C. trachomatis *progenitor.

**Conclusion:**

The evolutionary pathways of the chlamydial genome and plasmid imply that inheritance of the plasmid is tightly linked with its cognate chromosome. These data suggest that the plasmid is not a highly mobile genetic element and does not transfer readily between isolates. Comparative analysis of the plasmid sequences has revealed the most conserved regions that should be used to design future plasmid based nucleic acid amplification tests, to avoid diagnostic failures.

## Background

*Chlamydia trachomatis *is the most common cause of non specific urethritis in the industrialised world, and the major infectious cause of preventable blindness (trachoma) in the third world [1,2]. *C. trachomatis *can be divided into at least 15 serovars or serotypes, distinguished by *ompA *sequencing [3], which are associated with different disease pathologies [4]. Serotypes A, B, Ba and C are generally associated with blinding trachoma and serotypes D to K cause non-disseminating sexually transmitted infections. These 12 serotypes are all naturally restricted to infection of genital or ocular epithelial cells and have not been observed to be invasive [5]. By contrast, serotypes L1, L2 and L3 cause a rare invasive and systemic sexually transmitted infection normally found in the tropics, known as lymphogranuloma venereum (LGV) [6,7].

In October 2006 a new variant of *C. trachomatis *was described in Sweden that evaded several of the then current commercial molecular diagnostic tests for detecting the microorganism, which were based on the presence of specific plasmid sequences [8]. A deletion of 377 bp of plasmid DNA, in the region used for nucleic acid amplification tests (NAATs), is responsible for the negative diagnoses [9]. All new variant strains of *C. trachomatis *belong to serotype E [10]. Failure to detect the plasmid and hence treat those infected with the new variant has led to a significant increase in cases, and in some Swedish counties 20 – 64% of current infections are caused by this strain of *C. trachomatis *[10].

Strains of *C. trachomatis *have a highly conserved small genome of approximately 1 Mb and harbour a plasmid of approximately 7 kb [11]. Some plasmid-free isolates of *C. trachomatis *have been described, but these are exceedingly rare and the only viable clinical isolates described that are plasmid free belong to serotypes L2, D and E [12-14]. The *C. trachomatis *plasmids sequenced to date each contain eight predicted coding sequences (CDSs), along with a set of four 22 bp repeats which is understood to be the origin of replication [15,16]. Characterization of plasmid functions and of chlamydial genes in general has been greatly impeded by the lack of a genetic system for studying *C. trachomatis *[17] and so little is known about the function of these CDSs beyond *in silico *predictions of function for 5 CDSs and the observation that the CDS previously designated ORF5 encodes a protein of 28 kDa (pgp3) when expressed in *Escherichia coli *[18,19]. However, the role of pgp3 in the chlamydial developmental cycle and the overall biological function of the plasmid remains unknown.

Since the study of *C. trachomatis *genes is hampered by the lack of any molecular tools with which to manipulate strains, the emergence of the new variant strain and naturally occurring mutants provides an opportunity to investigate the evolution of the plasmids and to determine whether the evolutionary pathway of these plasmids matches that of the chromosome. In this study the nature and extent of chromosome and plasmid variation in *C. trachomatis *has been investigated. To extend the current dataset, two isolates of the previously unsequenced serotype B were completely sequenced, as were four plasmids from Swedish strains belonging to serotypes E and F, including that of the new variant strain. The plasmid is useful as a target for diagnostic testing because it is relatively stable and hence more resistant to nuclease damage than the genome, and present at up to ten copies per genome [20]. Thus a detailed knowledge of the sequence conservation and the stability of chlamydial plasmids will be critically informative in determining whether the plasmid is a reliable target for future diagnostic tests.

## Results and Discussion

### Genome sequences of serotype A and B isolates of *C. trachomatis*

The genomes of two serotype B ocular isolates of *C. trachomatis *were sequenced. These provide the first high quality genome sequences of ocular isolates: B/TZ1A828/OT and B/Jali20/OT (referred to as CTB and Jali20 respectively, for ease of nomenclature), summarised in Table 1. Whole genome comparisons showed that the genomes are highly syntenic, and that there are no whole gene differences between the strains. The pairwise mean nucleotide identity between orthologous genes is 99.8%. A comparison of the genomes against that of *C. trachomatis *serotype A strain A/HAR-13 (referred to as A/HAR-13) sequenced by microarray [21] showed no insertions or deletions of predicted coding sequences (CDSs).

**Table 1:** General properties of *C. trachomatis *genomes sequenced.

**Strain**	**B/TZ1A828/OT**	**Jali20**
Chromosome (bp)	1044282	1044352
% G+C	41.3	41.3
No. CDSs	879	875
Coding percentage	88.5	88.5
Average gene size (bp)	1051	1056
No. pseudogenes	14	18

The SNP comparisons have been made to the serotype A reference strain A/HAR-13.

Previous microarray and sequencing studies have shown that almost all sequence and gene variation distinguishing serotypes of *C. trachomatis *from each other are restricted to a region at the terminus of replication known as the Plasticity Zone (PZ) [22-24]. It was demonstrated by microarray and PCR analysis that a strain belonging to serotype B lacked most of the genes found within this region [22,23]. However, analysis of the serotype B isolates sequenced for this study showed that they possess many of the CDSs found within the PZ (see additional file 1). These include cytotoxin gene fragments, remnants of a much larger cytotoxin gene which is thought to have been similar to those still found intact in *C. muridarum *[25]. In a strain of *C. trachomatis *serotype D, a remnant of the cytotoxin gene is expressed, and is cytotoxic on HeLa cells [26]. Consistent with previous analysis of the PZ, the phospholipase D genes display high levels of disruption in both serotype B strains [24]. The PZ also encompasses the *trpRBA *operon, which has been found to be functional only in genital isolates, and to carry mutations in ocular strains [27]. This operon is non-functional in both the strains presented here, with either the *trpB *(B/TZ1A828/OT) or *trpA *(B/Jali20/OT) being disrupted (see additional file 1).

Despite there being no whole gene differences between the strains, analysis of the genomes identified several pseudogene differences when they were compared to each other and also with the genomes of other *C. trachomatis *serotypes and biovariants A/HAR-13 and the serotype L2 strain L2/434/BU (see additional file 2). Eleven of the disrupted CDSs are common to both serotype B strains, whereas only one of these is common also to A/HAR_13 and L2/434/BU (CTB_3211/JALI_3211/CTA_0350/CTL0578). The equivalent putative membrane proteins CTB_0571/JALI_0571 contain different inactivating mutations: a single base deletion causing a frameshift after codon 333 (CTB), or a single nucleotide polymorphism (SNP) prematurely truncating the protein after codon 177 (Jali20). Another example of a CDS with distinct inactivating mutations is the putative exported protein JALI_1341/CTA_0142. This CDS carries a frameshift mutation in Jali20, and is truncated by a premature stop codon leading to a loss of 60 amino acids from the C terminus in A/HAR-13.

Inclusion proteins are an important family of chlamydial proteins, associated with virulence, which target the host inclusion membrane. Consequently, the inactivation of CDSs CTB_2231 and JALI_2231 encoding candidate inclusion membrane proteins may have implications with regard to how the cell interacts with the host. This CDS has been disrupted by an identical SNP creating a stop codon after codon 113 in both strains, with JALI_2231 having undergone a further single base insertion and deletion at other sites to create two additional frameshifts. Another notable difference is the variation in the sequence of the *secF *gene, which is present as a full length gene in the serotype B strains, but is found as two separate CDSs *secD*/*secF *in A/HAR-13 [21]. A further functional loss is the operon comprising pyruvoyl-dependent arginine decarboxylase and arginine/ornithine antiporter, involved in pH homeostasis. The antiporter (CTB_3721/JALI_3721/CTA_0406-7) is disrupted in the serotype A and B strains, the decarboxylase (CTL0627) is disrupted in L2/434/BU, whereas the operon is intact in the serotype D strain D/UW-3/CX [24].

Set against a high level of sequence identity (generally in excess of 99% identity at the nucleotide level), some predicted CDSs display higher levels of variation (see additional file 3). As has been noted before, these include *ompA*, which is used to distinguish between serotypes [3], *tarp*, encoding the translocated actin recruiting phosphoprotein [24], and *hctB*, encoding histone-like HC2 [28].

### Phylogenetic analysis of *C. trachomatis *genomes

**Figure 1:**
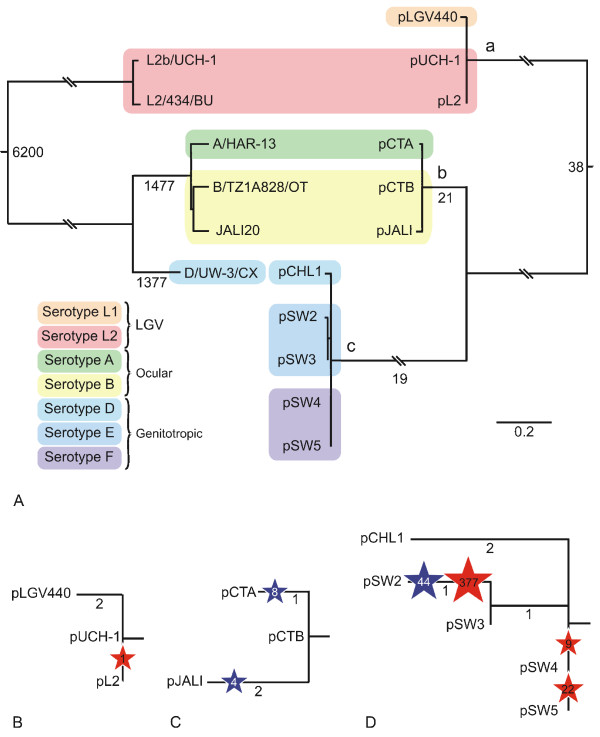
**Phylogenetic relationships within *C. trachomatis***. (A) Comparison of tree topologies produced by maximum likelihood analyses of plasmid (right) and full genome (left) sequences. Colours differentiate serotypes, as shown in the key. Branch lengths represent substitutions per site (see scale bar). Breaks in branches are equivalent to 0.4 substitutions per site. Numbers on nodes of plasmid tree represent numbers of SNPs occurring on that branch. Letters a-c indicate clades that are shown in more detail in B-D. (B-D) Detailed views of changes within clades a, b and c. Numbers on nodes represent numbers of SNPs occurring on that branch. Red stars represent deletions and blue stars represent insertions, with numbers inside the stars showing their length in bp.

The first genome-scale, SNP-based phylogenetic analysis of all six available *C. trachomatis *genomes was carried out, covering serotypes A, B, D, L2 and L2b (Table 2). Comparative genome analysis identified 11,500 SNPs, of which the large majority define splits between the three major groups (Figure 1). Monophyly of LGV strains is supported by 6200 SNPs, 1477 SNPs unite the three ocular strains and 1377 are unique to the genital, serotype D strain. These splits are also strongly supported in the results of the phylogenetic analysis of the SNPs, using a general time reversible model of evolution and four discrete gamma distributed rate categories to account for among site rate variation. Pairwise comparisons of the genomes by SNP numbers also confirms this clustering (Table 3). Within the ocular strains, the two serotype B isolates cluster together, suggesting that serotypes are identifiable on the basis of SNP phylogenies. These data are derived from the genomes of six isolates (five serotypes), whilst the numbers are small they reflect the same patterns of genome evolution observed using fragments of the genome [5] and our data using complete genomes are strong enough to support these associations, although further studies would be beneficial to confirm these findings when the technology will allow rapid easy purification of genomes from these obligate intracellular pathogens.

**Table 2:** Strains of *C. trachomatis *used in the study.

**Strain**	**Origin**	**Site**	**Serotype**	**Genome accession or [ref]**	**Plasmid**	**Plasmid size (bp)**	**Plasmid accession**
A/HAR-13	Saudi Arabia	conjunctiva	A	EMBL:CP000051	pCTA	7,510	EMBL:CP000052
Jali20	The Gambia	ocular	B	EMBL:FM872307	pJALI	7,506	EMBL:FM865438
B/TZ1A828/OT	Tanzania	ocular	B	EMBL:FM872308	pCTB	7,502	EMBL:FM865437
D/UW-3/CX	USA	cervix	D	EMBL:AE001273	N/A	N/A	N/A
G0/86	Italy	urethritis	D	[13]	pCHL1	7,502	Genbank:NC_001372
Sweden2	Sweden	male urethra	E	N/A	pSW2	7,169	EMBL:FM865439
Sweden3	Sweden	cervix	E	N/A	pSW3	7,502	EMBL:FM865440
Sweden4	Sweden	cervix	F	N/A	pSW4	7,493	EMBL:FM865440
Sweden5	Sweden	cervix	F	N/A	pSW5	7,471	EMBL:FM865442
L1/440/LN	USA	lymph node	L1	[4]	pLGV440	7,500	EMBL:X06707
L2/434/BU	USA	bubo	L2	EMBL:AM884176	pL2	7,499	EMBL:X07547
L2b/UCH-1	England	proctitis	L2b	EMBL:AM884177	pUCH-1	7,500	EMBL:AM886279

N/A – data not available.

**Table 3:** Pairwise comparison of SNP numbers between complete genome sequences.

	**L2b/UCH-1**	**L2/434/BU**	**A/HAR-13**	**B/TZ1A828/OT**	**Jali20**
L2/434/BU	487				
A/HAR-13	8688	8682			
B/TZ1A828/OT	8597	8587	1326		
Jali20	8561	8554	1256	1151	
D/UW-3/CX	8173	8139	3690	3669	3581

### Phylogenetic analysis of *C. trachomatis *plasmids

The plasmid sequences from the strains CTB and Jali20 were assembled from the genome shotgun. To investigate the new variant strain which evaded diagnosis, isolates from epithelial sexually transmitted infections (STIs) from the city of Malmo in Sweden were included. Genital tract isolates representing the new variant *C. trachomatis *(strain Sweden2, serotype E) and three concurrently isolated strains (Sweden3, serotype E; Sweden4 and Sweden5, both serotype F) were selected. To represent plasmids from other chlamydial serotypes, plasmid sequences were obtained from Genbank covering further trachoma and LGV strains (Table 2).

**Figure 2:**
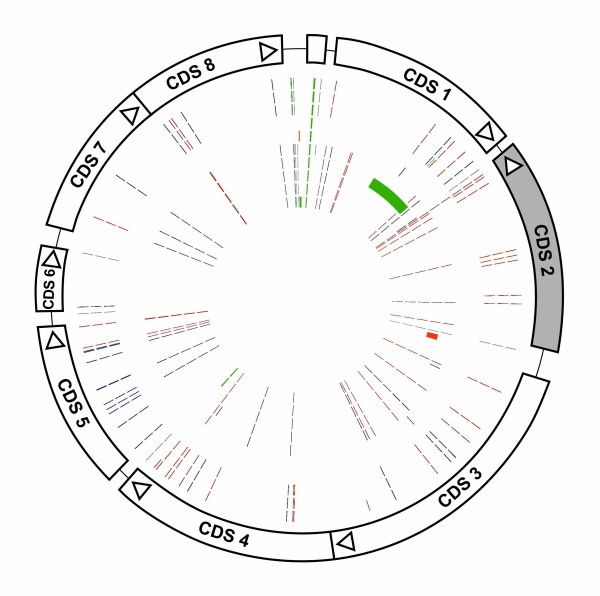
**Schematic of the location of SNPs and indels within the chlamydial plasmids**. The strain represented on the outer circle is plasmid pCTA from strain A/HAR-13. The positions of the CDSs are shown, with all CDSs transcribed in a clockwise direction except CDS2. The concentric circles represent (outer to inner): pLGV440, pUCH-1, pL2, pCTB, pJALI, pCHL1, pSW2, pSW3, pSW4, pSW5. The red lines indicate synonymous SNPs, the blue lines non-synonymous SNPs and the grey lines intergenic SNPs. The green bars are bases deleted in that plasmid, and the orange bars are regions of insertion/duplication.

Alignment of these 11 plasmid sequences showed that there are 83 SNP locations, representing approximately 1.1% variation. Six of these occur in intergenic locations. The SNPs and their effects on coding sequences are shown in Figure 2. Each plasmid is unique and identifiable by the presence of at least one SNP and/or indel (Figure 1B–D). Only two SNPs, at positions 5,328 and 7,458 (using pSW3 as the reference sequence), allow differentiation of the chlamydial plasmids into LGV, trachoma and genital tract groupings. Most of the SNPs are located within CDSs and there is no significant clustering of SNPs within the plasmid. Only one non-synonymous mutation occurs within CDS2 which may suggest that this gene is under strong selection. Analysis of the informative SNPs and indels allowed phylogenetic reconstruction of the relationships between the plasmids (Figure 1). The resulting phylogenetic tree shows that the chlamydial plasmids segregate into tight groupings reflecting the phenotypes of their host bacteria. The LGV plasmids are the most distantly related, with the STI and ocular strain plasmids having apparently diverged at a later time.

### Comparative phylogenetics of complete genomes and plasmids

A comparison of the phylogenies of the genomes and plasmids is given in Figure 1. Of the serotype A, B, L2 and L2b strains, for which both complete genome and plasmid sequence sets exist, and serotype D for which there are independent complete genome and plasmid sequences, the phylogenies mirror each other. The complete genomes and plasmids from the LGV strains cluster tightly, as do those from the ocular strains, and the STI strains branch from these and also cluster together.

These data suggest that the chlamydial plasmids have not been freely exchanged, but have remained closely linked to their cognate host chromosome (Figure 1).

### Analysis of the new variant plasmid

**Figure 3:**
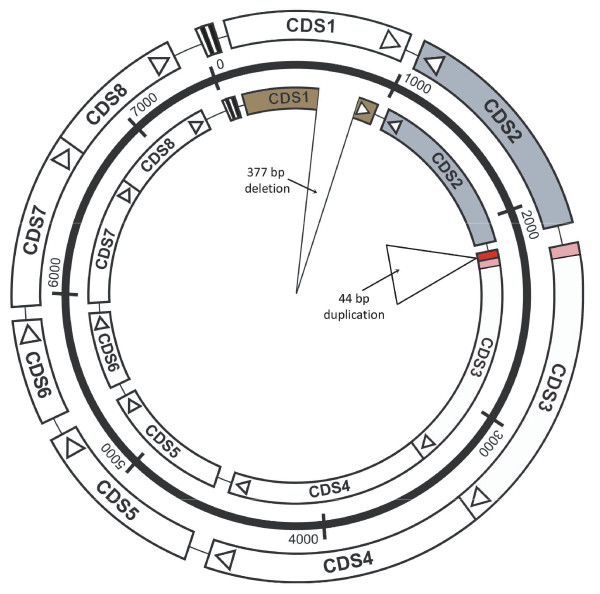
**Comparison of plasmids pSW2 (inner circle) and pSW3 (outer circle)**. pSW2 carries a 377 bp deletion in CDS1 (coloured brown for pseudogene) and a 44 bp duplication immediately upstream of CDS3 (shown in red). CDS2 is transcribed in the opposite direction to the other CDSs and is shaded grey. The set of 22 bp repeats upstream of CDS1 form the putative origin of replication.

The plasmid with the most variation (pSW2) was found in strain Sweden2. However, pSW2 still belongs to the genital tract lineage and therefore has not appeared as result of a transfer event. The complete nucleotide sequence of pSW2 comprises 7,169 bp, 333 bp smaller than the 7,502 bp plasmid (pSW3) from strain Sweden3. Sweden3 could be hypothesised to be the potential progenitor strain of Sweden2 because they have identical sequences of the chromosomal *ompA *gene. The difference in size between pSW2 and pSW3 is accounted for by a deletion of 377 bp and a duplication of 44 bp at a different locus (Figure 3). pSW2 is the smallest chlamydial plasmid described, some 200 bp smaller than the previously smallest known chlamydial plasmid, pCpnE1 (7,369 bp), which is from an equine strain of *C. pneumoniae *[29,18].

The 377 bp deletion within pSW2 is situated within CDS1, creating a frameshift which shortens the predicted protein from 305 to 178 amino acids and removing a primer binding site for the diagnostic nucleic acid amplification tests (NAATs). This region of the plasmid was originally selected as the target for several commercial diagnostic NAATS and the new variant strain became established because infection by this strain went undetected and hence untreated [9]. Plasmid pL2 from strain L2/434/BU contains a single nucleotide deletion within CDS1 (position 910) [15,30] leading to a truncated CDS1 protein (260 amino acids). pCpnE1 also has a deletion within CDS1, but the location is different to that within pSW2 and the effect is to create two small putative CDSs, which are unlikely to be functional.

The observation of CDS1 as a region of the plasmid apparently prone to inactivation and therefore potentially dispensable may be explained by the possible functional redundancy between the proteins encoded by CDS1 and CDS2. These proteins have similar sizes (305 and 332 amino acids respectively), share 35% amino acid sequence identity, and both match to the Pfam domain PF00589 (CDS1 e-value of 0.003, CDS2 e-value of 4.9e-39), suggesting some functional equivalence [29].

A second difference between the pSW2 and the other *C. trachomatis *plasmids is a 44 bp perfect tandem duplication, located immediately upstream of both CDS2 and CDS3, which are divergently transcribed. The transcription start points (tsp) for CDS2 and CDS3 (encoding a homologue of DnaB, a protein involved in forming the replication complex) have been mapped previously [31] and are both located within this 44 bp section. The duplication of the tsp could potentially boost CDS2 expression. Both the deletion in CDS1 and the 44 bp duplication are unique to pSW2, and no intermediate plasmid carrying either of the mutations separately has been identified. This could indicate that these changes are related events, and that potential up-regulation of CDS2 may compensate for the loss of a functional product from CDS1.

### Candidate plasmid regions for improved diagnostic targets

When all *C. trachomatis *plasmid CDSs from the eleven complete nucleotide sequences were compared, CDS2 was found to be the most highly conserved. Although there are eleven SNPs within the coding sequence, only one results in an amino acid change (Figure 2), suggestive of a functional requirement. This SNP (Met-Leu, position 1,147), present in pSW2 and pSW3, is at the extreme carboxy terminus of the protein. A further constraint on variation within CDS2 is the presence of two short RNA molecules (225 and 415 nucleotides), which are complementary to the 3' terminus of the primary transcript encoding CDS2 [32]. These two short 'antisense' transcripts are differentially expressed during the developmental cycle. This level of sequence conservation, possibly tied to an essential function, suggests that the region of the plasmid encompassing CDS2 would be a good target for future screening.

CDS6, CDS7 and CDS8 also show high levels of amino acid conservation. CDS6 (unknown function) is the smallest plasmid encoded protein and contains a single SNP. The proteins encoded by CDS7 and CDS8 display homology to proteins involved in the process of plasmid partitioning [29], and have been shown to be active at cell division. These proteins may play an important role for these relatively low copy number plasmids, ensuring that each daughter cell acquires an equal number of plasmid copies.

The protein predicted to be encoded by CDS5, previously designated ORF 5 (pgp3) has the largest number of non-synonymous SNPs. There are 14 SNPs, evenly spread throughout CDS, resulting in ten amino acid changes (Figure 2). SNP 5,112 differentiates LGV plasmids from the trachoma plasmids and SNP 5,114 is unique to the blinding trachoma isolates (using pSW3 as the reference sequence). The protein encoded by CDS5 (pgp3) has been located to the cell surface and it has recently been suggested that the CDS5 product can be secreted from inside the inclusion, to the cytoplasm of *Chlamydia*-infected cells [33]. Thus the higher number of non-synonymous changes in this CDS could result from immune selection giving rise to more variation.

The area of the plasmid from the stop codon of CDS8 to the start codon of CDS1 has the highest density of intergenic SNPs, as well as apparent deletions, making the region the most susceptible to mutation within the *C. trachomatis *plasmids. Thus the area around the replication origin is the most variable and is a poor region in which to design diagnostic PCR primers.

### Analysis of plasmid copy number

**Figure 4:**
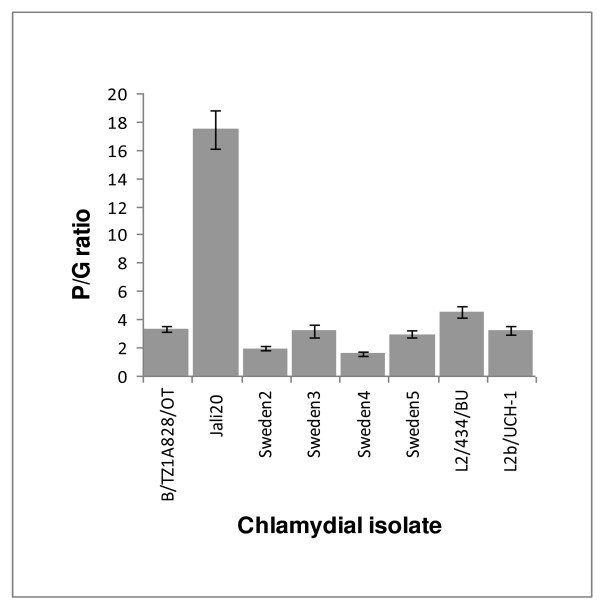
**Plasmid copy number in *C. trachomatis***. Quadruplicate samples of chromosomal DNA purified from EBs were analysed by qPCR to determine the plasmid/genome ratios (P/G).

The sequencing of pSW5 revealed that this plasmid carries one 22 bp repeat fewer than the others at the putative origin of replication. To test whether this affects plasmid copy number, DNA from several strains was subjected to quantitative PCR. The results showed that, where loss of the repeat sequence had occurred, plasmid copy number was not adversely affected, with plasmid/genome (P/G) ratios in the range of 2–6 (Figure 4). Interestingly, it appears that genital strains have a slightly lower plasmid copy number than the others, and strain Jali20 has the highest P/G ratio.

## Conclusion

The chromosomes and plasmids of *C. trachomatis *exhibit extreme levels of sequence conservation and co-inheritance. This may be accounted for by the constraints demanded by the obligate intracellular developmental cycle, and the lack of a route for foreign DNA exchange. Attempts to cure *C. trachomatis *of its plasmid using chemical agents have been unsuccessful [20], and viable plasmid-free isolates of *C. trachomatis *(plasmid extinction) are exceedingly rare, with only three such isolates reported. The plasmid is therefore not essential for chlamydial viability, but a recent study comparing the pathobiological properties of a naturally occurring plasmid-free L2 isolate with a plasmid-bearing strain suggested that the plasmid may have a role in virulence [34]. Furthermore, studies with plasmid cured *C. muridarum *showed that this strain failed to cause disease within the infected mouse oviduct [35].

A more detailed examination of the plasmids and genomes of *C. trachomatis *was prompted by the appearance and spread of a new variant strain in Sweden, carrying significant changes to its plasmid. Previous phylogenetic analyses of chlamydial plasmids indicated that their evolutionary lineage includes plasmids from other chlamydial species [29]. Complete genome SNP analysis of the *C. trachomatis *genomes and plasmids presented here indicates that the plasmids from the lymphogranuloma biovars diverged from the trachoma biovars first and this has been followed by subsequent evolution of these plasmids following biological pathways of ocular or genital strains. The current data set therefore provides no evidence that there has been any exchange of plasmids between ocular, genital tract and LGV serovar groupings. There is no accurate molecular clock to measure the evolutionary rate within *C. trachomatis*, but as these are exclusively human pathogens it would seem likely that these events have occurred within recent human evolution. The discovery of the new variant *C. trachomatis*, undocumented before 2002, strongly suggests either that the novel mutations within the plasmid are very recent [10], or that this variant has become prominent due to the reliance on a plasmid-based diagnostic test.

The cryptic plasmid is an important target for chlamydial detection and knowledge of its structure and variation is essential in designing accurate and sensitive new assays. The data presented here indicate that the CDS2 region of the plasmid is the most highly conserved part of the sequence and thus the optimal choice for developing new nucleic acid amplification tests. Each plasmid analysed has a unique sequence, reflecting its associated serotype; however the differences between some are very slight and it is unlikely that a useful typing system could be developed based on the plasmid alone. Undoubtedly, the changes in the new variant *C. trachomatis *plasmid give it a distinctive signature which will allow its spread in new populations to be followed. Even with these large differences between the new variant plasmid pSW2 and its putative progenitor pSW3, it is clear that the chlamydial plasmid remains closely associated with its cognate chromosomal partner. The parallel evolution of the chlamydial plasmid and chromosome, and lack of horizontal gene transfer, is consistent with the intracellular developmental cycle of this species.

## Methods

### Chlamydia and cell culture

The isolates and sources of *Chlamydia trachomatis *used in this work are summarised in Table 2. Clinical samples of *C. trachomatis *Sweden 3–5 were isolated in Malmo, Southern Sweden in 2002, and Sweden 2 was obtained in 2006. They were isolated in McCoy cell cultures by removing 100 μl of eluate from the original swab and direct inoculation onto a glass coverslip within a bijou containing Dulbecco's modified Eagles' medium (DMEM) plus 0.03 M glucose, vancomycin at 10 μgml^-1 ^and gentamicin 10 μgml^-1^. The inoculae were centrifuged onto the cell cultures at 1,000 g for 1 hr at room temperature. Following centrifugation the cell culture supernatant was removed and cycloheximide-containing medium added to the infected cells which were incubated in 5% CO_2 _for 3 days. Viable *C. trachomatis *cells were observed by phase contrast microscopy.

Strains of *C. trachomatis *Sweden 2–5 and B/Jali20/OT [36] were negative when tested for mycoplasma contamination by fluorescence microscopy using Hoechst no. 33258 staining and by using a VenorGem^® ^Mycoplasma PCR Detection Kit (Minerva Biolabs, Berlin, Germany) according to the manufacturer's instructions. The McCoy cells and BGMK cells (used for LGV isolates) were grown in Dulbecco's modified Eagles' medium (DMEM) supplemented with 10% foetal calf serum. Infected monolayers were detached with PBS containing 0.125% trypsin/0.02% EDTA and elementary bodies (EBs) were harvested in DMEM containing 10% FCS at 3,000 × g for 10 min. The infected cell pellet was suspended in PBS:H_2_O (1:10) and homogenised in a Dounce homogeniser to break open cells and release the EBs. Cell debris was removed by centrifugation at 250 g for 5 min and the supernatant containing partially purified chlamydiae was mixed with an equal volume of phosphate buffer containing 0.4 M sucrose (2SP), prior to storage at -80°C.

### Preparation of chlamydial DNA and quantitative PCR

Large scale preparations of each *C. trachomatis *isolate were prepared and EBs were purified as described [37]. For the non LGV strains confluent monolayers of cells in 75 cm^2 ^flasks were infected by centrifugation at 1,000 g for 1 hr. Chromosomal DNA was extracted from gradient purified EBs using a Promega Wizard Genomic Purification kit according to the manufacturer's protocol. The only modification was that purified EBs were incubated with proteinase K for 1 hr at 60°C prior to addition of the Nuclei lysis solution. The quality of genomic and plasmid DNA recovered by this method was assessed by agarose gel electrophoresis. An accurate determination of plasmid and genome copies for each DNA preparation was obtained by performing 5'-exonuclease (TaqMan) assays [20]. The primers and probes have been described previously and were selected from conserved DNA sequence regions from the databases: CDS2 from the plasmid and the chromosomal *omcB *gene.

### DNA sequencing and analysis

The genomes of *C. trachomatis *strains B/TZ1A828/OT [38] and B/Jali20/OT [36] were sequenced to a depth of 8 × coverage derived from pUC18 (insert size 1.4–2.2 kb) small insert libraries using dye terminator chemistry on ABI3700 automated sequencers. End sequences from larger insert plasmid (pMAQ1 9–12 kb 9–12 kb insert size) libraries were used as a scaffold. Plasmid sequencing was performed using custom primers (see additional file 4). Plasmid DNA was amplified using Platinum^® ^Pfx DNA polymerase (Invitrogen) and primer pairs 295r/460f (product size 5 kb), 1200r/6761f (1.6 kb), 460f/3650r (3.3 kb), 6470f/5820r (2.3 kb) and ctp6/1200r (3.5 kb). All sequences were subject to sequencing, assembly, finishing and checking as described [39], with annotation facilitated by the programs Artemis [40] and ACT [41]. Pseudogenes were curated manually, and the original sequence data double checked, with truncations and frameshifts determined through comparison with other published *C. trachomatis *genomes. Plasmid CDSs with the locus_tag XXXX_1 are referred to in the text as CDS1, etc. The genome and plasmid sequence accession numbers are listed in Table 2.

### Phylogenetic analyses

Plasmid and genome DNA sequences were aligned using MUSCLE v3.52 [42] and the progressive algorithm of Mauve [43] respectively, using default parameters. Invariant sites were removed from the alignments, and an appropriate model of evolution was identified using the Akaike information criterion within Modeltest [44]. Maximum likelihood analysis of each alignment was carried out in RAxML v7.0.0 [45] using this model (GTR+gamma with 4 substitution rate categories).

## Authors' contributions

HMBS-S conceived, designed and performed experiments, performed annotation and helped write paper. SRH analysed and interpreted data and helped write paper. KP contributed reagents/mats/analysis tools and conceived experiments. PM analysed data, conceived and designed experiments and helped write paper. CB contributed reagents/mats/analysis tools and conceived experiments. LTC conceived and performed experiments. PRL conceived experiments, analysed data and helped write paper. SJL conceived and performed experiments. OS conceived and performed experiments. RJS conceived and performed experiments. YW conceived and performed experiments. MJH contributed reagents/mats/analysis tools. JP conceived and designed experiments and helped write paper. NRT conceived and designed experiments and helped write paper. INC conceived and designed experiments and wrote paper. All authors read and approved the final manuscript.

## Supplementary Material

Additional file 1 **Comparison of the Plasticity Zone between several strains, visualised by the Artemis Comparison Tool**. The grey lines indicate forward and reverse reading frames of sequenced genomes, with predicted coding sequences superimposed. The red bars indicate regions of 97–100% nucleotide identity. Brown CDSs denote pseudogenes. The cytotoxin locus is reduced in D/UW-3/CX, yet produces an active cytotoxin. It is further deleted in strain L2/434/BU. The phospholipase D locus contains pseudogenes in all strains. The *trp *operon is complete in strains D/UW-3/CX and L2/434/BU, but has pseudogene components in the serotype A and B strains: *trpB *in B/TZ1A828/OT and A/HAR-13, and *trpA *in Jali20 and A/HAR13. Click here for file

Additional file 2 **Pseudogene differences between strains B/TZ1A828/OT, B/Jali20, A/HAR-13 and L2/434/BU**. Pseudogenes (Ψ) are highlighted in brown, and the CDSs contained within the plasticity zone are shown in mauve. Click here for file

Additional file 3 **Variable CDSs, comparing strains B/TZ1A828/OT, B/Jali20 and L2/434/BU**. CDSs with significant variability between the strains are listed, with brief description of variation. Click here for file

Additional file 4 **Custom primers used for amplification and sequencing of *C. trachomatis *plasmids**. Click here for file
