# Supplementary figures and images for: Co-evolution of genomes and plasmids within Chlamydia trachomatis and the emergence in Sweden of a new variant strain
Source: BMC Genomics. 2009 May 21;10:239. doi: 10.1186/1471-2164-10-239 (PMC2693142; doi:10.1186/1471-2164-10-239)

## Slide 1
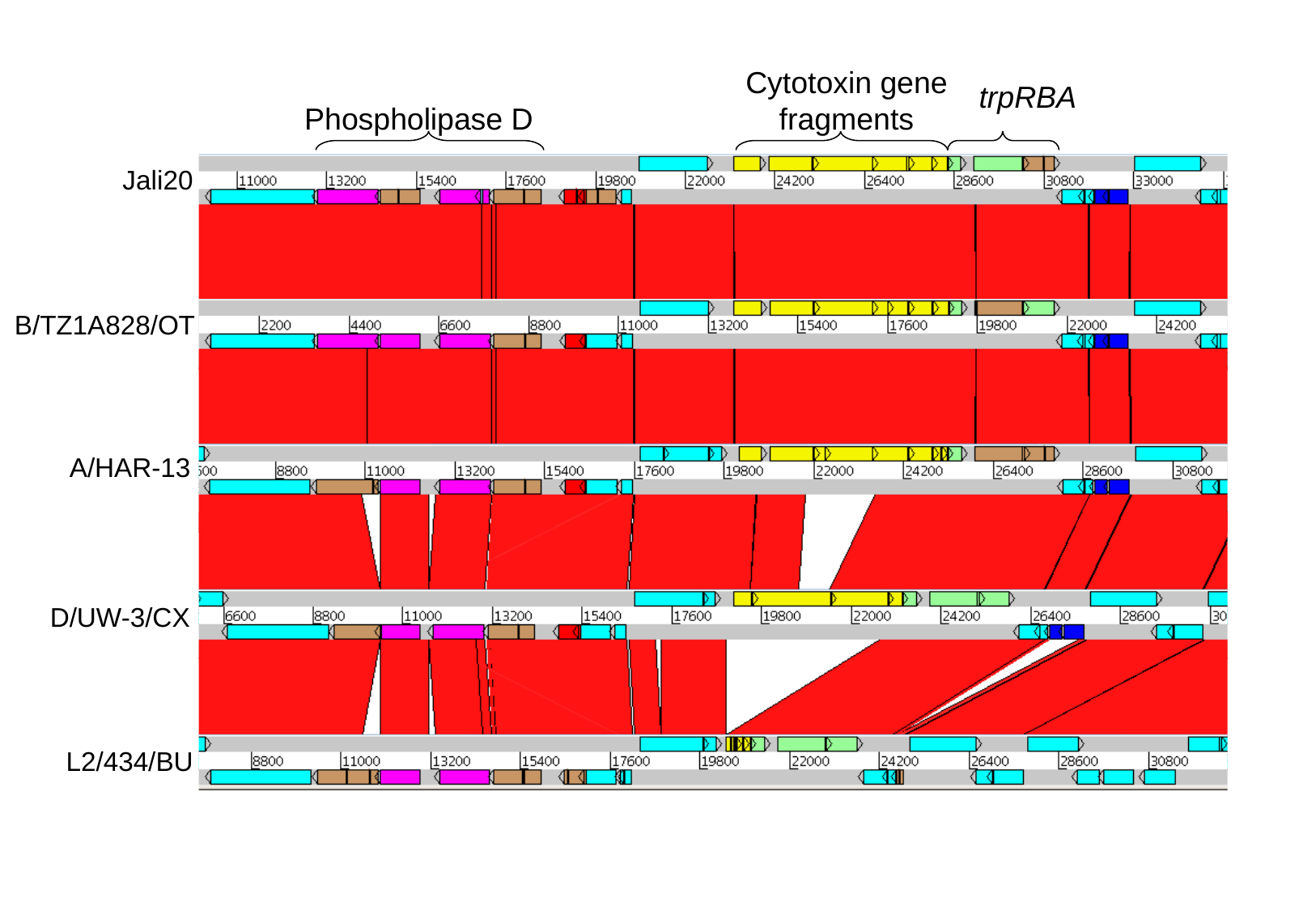

Cytotoxin gene fragments
trpRBA
Phospholipase D
Jali20
B/TZ1A828/OT
A/HAR-13
D/UW-3/CX
L2/434/BU

Supplement: Additional file 1 — Comparison of the Plasticity Zone between several strains, visualised by the Artemis Comparison Tool. The grey lines indicate forward and reverse reading frames of sequenced genomes, with predicted coding sequences superimposed. The red bars indicate regions of 97–100% nucleotide identity. Brown CDSs denote pseudogenes. The cytotoxin locus is reduced in D/UW-3/CX, yet produces an active cytotoxin. It is further deleted in strain L2/434/BU. The phospholipase D locus contains pseudogenes in all strains. The trp operon is complete in strains D/UW-3/CX and L2/434/BU, but has pseudogene components in the serotype A and B strains: trpB in B/TZ1A828/OT and A/HAR-13, and trpA in Jali20 and A/HAR13. [file 1471-2164-10-239-S1.ppt]
